# 5-Aminolevulinic acid-mediated protoporphyrin IX accumulation in the tumor microenvironment of glioma and its implications for 5-aminolevulinic acid-based local tumor therapies: A review of the literature

**DOI:** 10.1093/neuonc/noag094

**Published:** 2026-04-30

**Authors:** Zeynep Özdemir, Marco Gallus, Michael Müther, Walter Stummer

**Affiliations:** Department of Neurosurgery, University Hospital Münster, Münster, Germany; Department of Neurosurgery, University Hospital Münster, Münster, Germany; Department of Neurosurgery, University Hospital Münster, Münster, Germany; Department of Neurosurgery, University Hospital Münster, Münster, Germany

## Abstract

5-Aminolevulinic acid (5-ALA)-induced protoporphyrin IX (PpIX) accumulation is widely used for fluorescence-guided surgery in malignant glioma. Beyond its diagnostic role, PpIX exhibits photo-, sono-, and radiosensitizing properties that enable locally applied tumor therapies. For these modalities, the cellular and subcellular localization of PpIX is a critical determinant of therapeutic efficacy, as it defines the site of reactive oxygen species generation and subsequent biological effects. In this comprehensive literature review, the cellular and subcellular distribution of 5-ALA–induced PpIX in gliomas and their tumor microenvironment (TME) was assessed. Studies were identified through a structured MEDLINE search and evaluated for evidence of PpIX localization in neoplastic and nonneoplastic cellular components. Accumulating data indicate that PpIX localizes not only within malignant glioma cells but also across multiple cellular components of the TME. Advanced imaging, single-cell, and spatial transcriptomic analyses demonstrate PpIX fluorescence frequently aligns with immunosuppressive myeloid populations and infiltrative tumor regions. Subcellularly, PpIX localizes in mitochondria, lysosomal-autophagic compartments, and extracellular structures, in a cell-type dependent manner. These distribution patterns provide a biological basis for the immunomodulatory, antiangiogenic, and cytotoxic effects observed in studies of 5-ALA-mediated local therapies. While macroscopic fluorescence remains clinically valuable for fluorescence-guided resection, the biologically relevant microscopic distribution of PpIX supports the concept that PpIX-based therapies may target not only tumor cells but also immunosuppressive and stromal compartments. A refined understanding of PpIX localization is therefore central to optimizing nonsurgical 5-ALA-based therapies and their integration into multimodal glioma treatment paradigms.

Key Points

- Protoporphyrin IX (PpIX) accumulates in cellular and noncellular components of the tumor microenvironment (TME), including tumor-associated macrophages and microglia.
- Myeloid PpIX accumulation enables selective targeting using 5-aminolevulinic acid-based local therapies, which may support immunogenic TME modulation.

Glioblastoma (GBM) is the most prevalent primary malignant brain tumor with an incidence of 3/100 000.^1-4^ Prognosis is poor with a median survival of approximately 15 months.^1-4^ The mainstay of treatment involves maximal safe resection and adjuvant chemoradiation.^2^ The common standard in glioma surgery is fluorescence-guided surgery (FGS) using 5-aminolevulinic acid (5-ALA) which has demonstrated to improve the extent of resection and prolong progression-free survival resulting in its approval for malignant glioma surgery by the U.S. Food and Drug Administration (FDA) and European Medicines Agency (EMA).^5-11^ As a natural precursor of heme, after oral administration, 5-ALA selectively induces the accumulation of protoporphyrin IX (PpIX) in malignant glioma tissue that can then be excited during surgery with blue light in the 400 nm range resulting in pink fluorescence in tumor cells.^6^^,^^7^^, ^^12^ The tumor selective PpIX accumulation after 5-ALA administration is partly attributable to the disrupted blood-brain barrier (BBB) in high-grade glioma facilitating enhanced 5-ALA uptake and diffusion with edema into the peritumoral infiltration zone.^13^ Other mechanisms, such as active membrane transport, also contribute.^13^ Multiple studies have demonstrated that the PpIX fluorescence in FGS goes beyond the contrast-enhancing tumor.^8^^,^^9^ Apart from its use in FGS, PpIX has photo-, sono-, and radiosensitizing properties, enabling local tumor treatment modalities, such as photodynamic therapy (PDT), sonodynamic therapy (SDT), and radiodynamic therapy (RDT). Upon excitation with the appropriate energy source, that is, light in the 635 nm range for PDT, ultrasound for SDT, and ionizing radiation for RDT, PpIX generates reactive oxygen species (ROS) resulting in oxidative stress and cellular toxicity (see Figure 1). While for FGS the macroscopically perceived fluorescence is pivotal for surgical navigation and maximizing the extent of resection, the specific cellular and subcellular localization of PpIX is of limited relevance during surgery.^5^^,^^12^

**Figure 1.**
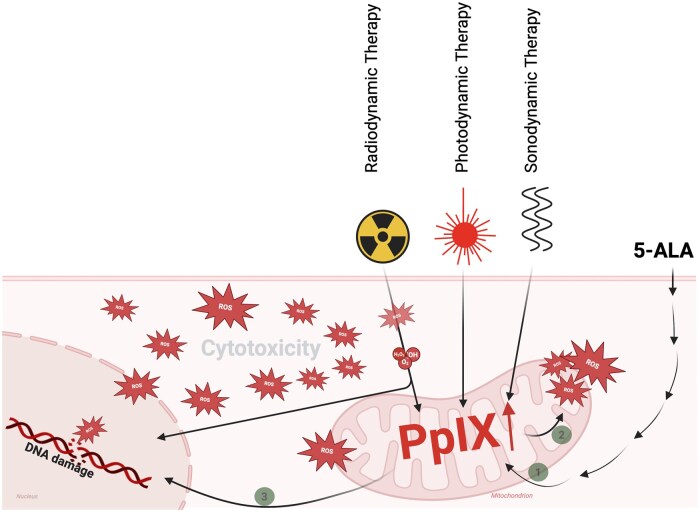
Schematic illustration of photo-, sono-, and radiodynamic properties of 5-ALA. Exogenously administered 5-ALA is taken up by tumor cells and metabolized through the heme biosynthesis pathway to PpIX. Due to altered enzymatic functions and dysregulated heme biosynthesis, PpIX accumulates mainly in the mitochondria (1). Excitation of PpIX with light in the 635 nm range (PDT) or ultrasound (SDT) results in ROS-mediated cytotoxicity (2). Ionizing radiation induces ROS via water radiolysis, while intracellular PpIX further amplifies ROS-generation creating a synergistic effect and resulting in mitochondrial dysfunction and increased oxidative DNA damage, including enhanced double-strand DNA breaks, thereby increasing overall cytotoxic effects (3). Abbreviations: PDT, photodynamic therapy; PpIX, protoporphyrin IX; ROS, reactive oxygen species; SDT, sonodynamic therapy; 5-ALA, 5-aminolevulinic acid. (Created in BioRender. Özdemir, Z. (2025) https://BioRender.com/jhrsfak.)

However, for local treatment modalities, namely, PDT, SDT, or RDT, the location of the sensitizing agent, that is, PpIX, defines the site of oxidative stress and therefore, overall therapy efficacy. With such therapies, the cell-specific localization of PpIX within the tumor microenvironment (TME) may play a key role in determining the mechanism of action and the efficacy of local nonsurgical treatment modalities. If PpIX accumulates not only in tumor cells but also in immunosuppressive cells of the TME, nonsurgical treatment approaches may be more effective than strategies that exclusively target tumor cells. To date, targeted therapies in glioblastoma have shown limited clinical benefit.^14^

## 5-ALA Metabolism and Selective PpIX Accumulation in Glioma

5-Aminolevulinic acid is metabolized via the heme biosynthesis pathway. Two molecules of 5-ALA condense to form porphobilinogen which is then converted through several enzymatic steps to urophorphyrinogen III and ultimately coproporphyrinogen III. Coproporphyrinogen III is then transported into the mitochondria via the ABCG6 transporter, where it is oxidized to protoporphyrinogen IX and ultimately to PpIX. Under physiological conditions, PpIX is chelated with ferrous iron (Fe^2+^) to heme.^15^ Although still not fully understood, the selective PpIX accumulation in glioma is considered to be a multifactorial phenomenon. One major determinant is the disrupted BBB.^16^ In GBM, tumor-associated neovascularization leads to the structural and functional loss of the BBB by producing leaky vasculature.^13^^,^^17^ This results in enhanced 5-ALA penetration into glioma tissue and, via perilesional edema, into the infiltration zone where cellular uptake mechanisms gain importance.^13^ A key mechanism underlying PpIX accumulation is the reduced ferrochelatase activity as well as reduced Fe^2+^ availability in GBM, impairing conversion to heme.^18-21^ Additionally, highly proliferative tumor cells also exhibit an altered and dysregulated heme biosynthesis pathway, including upregulation of several upstream enzymatic activity, such as ALA-dehydrogenase or porphobilinogen-deaminase, resulting in increased production of PpIX precursors.^22-25^ Elevated expression of transport proteins, such as PEPT1 and PEPT2, facilitating the transport of 5-ALA into the cell, also appear to contribute to tumor selectivity. However, for glioma, the strongest evidence only exists for PEPT2.^26-28^ Increased expression of ABCG6, the transporter of coproporphyrinogen III into the mitochondria, has been correlated with PpIX fluorescence in human glioma tissue samples suggesting an additional mechanism contributing to tumor selectivity.^21^ Conversely, ABCG2 is considered one of the principal regulators of intracellular PpIX level, as it actively pumps PpIX out. ABCG2 mediates efflux of PpIX from the mitochondria to the cytosol and subsequently across the plasma membrane. This transporter is highly upregulated in multiple cancers, contributing to multidrug resistance.^29^

## The Tumor Microenvironment and 5-ALA Induced Porphyrins

Glioblastoma is characterized by high intratumoral heterogeneity, and marked capacity for adaptive resistance and immune evasion, which is predominantly a result of its complex and highly immunosuppressive TME.^30^^,^^31^ The TME is comprised of a heterogenous population of immune cells, nonimmune cells, including endothelial cells, astrocytes, oligodendrocytes, and neurons, as well as noncellular components.^30^^,^^31^ Glioma cells modulate cellular and noncellular components of the TME through signaling pathways. Disruption of the BBB represents a central factor in the establishment and maintenance of this specialized TME.^30^^,^^31^ The BBB is comprised of endothelial cells, pericytes, and astrocytes, which together regulate vascular permeability. Glioma cells secrete, among others, VEGF and matrix metalloproteinases that comprise BBB integrity by increasing permeability through degradation of tight junctions, basement membrane components, and increasing endothelial cells fenestration.^30^^,^^31^ In particular, VEGF is a potent angiogenic factor that also promotes tumor-associated neovascularization, resulting in a highly irregular, leaky, and functionally compromised vascular network with abnormal vascular permeability. The increased permeability facilitates the infiltration of immune cells and the entry of carcinogenic factors into the TME, thereby supporting glioma development and progression.^30^^,^^31^ Tumor-associated macrophages (TAMs) constitute one of the predominant immune-cell population within the TME and are actively recruited by glioma-derived cytokines, such as CCL2.^30^^,^^31^ Rather than fitting a strict M1/M2 polarization paradigm, TAMs exist along a dynamic spectrum of activation states shaped by hypoxia, tumor-derived cytokines, metabolic stress, and therapeutic exposure. TAMs, particularly subsets enriched for immunoregulatory phenotypes contribute to immunosuppression through the secretion of anti-inflammatory cytokines, as well as factors that promote tumor growth and angiogenesis.^30-32^ Metabolically, TAMs within GBM exhibit distinct iron handling, mitochondrial activity and heme pathway modulation, that may influence intracellular PpIX retention. Microglia are also amongst the most abundant immune cells in the TME and secrete a range of growth factors and cytokines, including IL-6, TGF-ß, and VEGF, thereby, supporting tumor growth. Importantly, microglia play a key role in GBM resistance to radio- and chemotherapy.^30-32^ In contrast, adaptive immune cells, such as T cells and natural killer cells are comparatively scarce and frequently exhibit functional impairment due to the strongly immunosuppressive environment. T-cell dysfunction is primarily driven by tumor-induced T-cell fatigue, immunological checkpoint activation and the infiltration of regulatory T-cells, which collectively suppress effective anti-tumor immune responses and promote an immunosuppressive environment. B-cells are also part of the adaptive immune response and have been implicated in promoting tumor cell proliferation, migration and angiogenesis within the TME.^30^^,^^31^^,^^33^ Myeloid-derived suppressor cells constitute an additional immunosuppressive component of the TME and impair the function of immune effector cells. They further promote tumor growth through the secretion IL-6 and suppress natural killer cell activity via upregulation of programmed death-ligand 1 expression.^30^^,^^31^^,^^33^ Nonimmune cellular components of the TME include endothelial cells, which also contribute to tumor-associated angiogenesis, secrete immunosuppressive factors and also remodel the extracellular matrix, facilitating tumor cell migration. Reactive astrocytes also shape the TME by releasing cytokines, growth factors, and metabolites that promote tumor cell survival and local invasion.^30^^,^^31^

Studies increasingly demonstrate that exogenous 5-ALA administration not only results in PpIX accumulation in glioma cells but also in various cellular and noncellular components within the TME, including, microglia, macrophages, astrocytes, and vascular or stromal elements. This phenomenon is of limited relevance for FGS, as the perceived fluorescence primarily reflects overall tumor tissue PpIX concentrations and optical properties rather than cell-type specificity. However, PpIX uptake in cellular and noncellular components of the TME will enable addressing components with PpIX-based local therapies. Such treatment modalities have potential to reduce highly therapy-resistant immunosuppressive cell populations within the TME and consequently, promote a more immunogenic microenvironment.

The aim of this study is to provide a comprehensive review of the literature on the cellular and subcellular localization of PpIX in gliomas, with a particular focus on its implications for PpIX-mediated local tumor therapies. We synthesize available evidence on the distribution of 5-ALA–induced PpIX accumulation within the TME to establish a framework for identifying potential cellular targets for nonsurgical, locally active 5-ALA-based therapeutic strategies.

## Methods

A MEDLINE search in titles and abstracts was performed using the following search operators on November 3, 2025: (5-ALA OR 5-aminolevulinic acid) AND (tumor microenvironment OR TME OR subcellular OR localization OR single cell) AND (glioma OR GBM OR cancer OR tumor). Inclusion criteria comprised preclinical and clinical studies investigating the localization of PpIX following 5-ALA administration, including studies combined with local therapies. Studies primarily focusing on CNS tumors were included, with additional consideration given to relevant non-CNS tumor studies. Exclusion criteria included non-English publications, duplicates, and studies not addressing PpIX localization. Endnote X9 (Clarivate Analytics) was used as a search engine. Two reviewers independently performed the search (Z.Ö. and W.S.). This systematic review is reported in accordance with the Preferred Reporting Items for Systematic Reviews and Meta-Analyses Statement.

## Results

A total number of 6780 studies from 1995 to 2025 were identified. After removing duplicates and studies without abstracts, the titles, and abstracts of 5893 studies were screened. Full-text articles of 60 studies were assessed. Finally, 12 articles were identified that met all criteria and were included for qualitative analysis. Overall, 8 studies on brain tumors and 5 studies on other tumor entities were identified (see Table 1).

**Table 1.** Cellular and subcellular localization of PpIX following 5-ALA application

	Experimental model	PpIX localization
Cellular localization		
Pacioni et al^34^	Human HGG and LGG specimen	CRMP5-expressing malignant tumor cells
Andrieux et al^35^	Human GBM specimen	Mesenchymal subtype of GBM cells Malignant tumor cells Myeloid-lineage cells
Lang et al^36^	Human GBM specimen	CD8-, CD68-, CD168-, and FoxP3-positive cells
Liu et al^37^	Human GBM specimen In vitro and in vivo (TS543, HMC3 and GL261 cell lines)	Malignant tumor cells Oligodendrocytes Myeloid cells incl. microglia T-cells
Ramapriyan et al^38^	Human meningioma specimen	CD3- and CD68-positive cells
Nasir-Moin et al^39^	Human GBM specimen	CD163+ macrophages GFAP+ astrocyte-like cells Mesenchymal-like malignant cells TAMs Oligodendroglial cells
Subcellular localization		
Gaullier et al^40^	In vitro (NCTC keratinocytes)	Plasma membrane Lysosomal structures
Chen et al^41^	In vitro (JCS leukemia cell line)	Cytoplasm Mitochondria
Ji et al^42^	In vitro (esophageal carcinoma cell line KYSE-450 and KYSE-70**)**	Mitochondria Cytoplasm Plasma membrane
Chen et al^43^	In vitro (DHL lymphoma cell line)	Mitochondria Endoplasmic reticulum (ER) Lysosomal structures
Wang et al^44^	In vitro (S180 sarcoma cell line)	Mitochondria Cytoplasm Plasma membrane
Jones et al^45^	Human GBM-patient plasma samples In vitro and in vivo (Gli36 and HBMVEC cell lines)	Extracellular vesicle
Fredericks et al^46^	In vitro (U118MG cell line)	Autophagy-lysosomal compartment

### Cellular and Subcellular Localization of PpIX in Brain Tumors

#### Cellular localization

Six studies specifically focusing on cellular localization of PpIX in glioma were identified. Pacioni et al assessed PpIX fluorescence in human low-grade and high-grade glioma tissue specimen, demonstrating that 32.7%-75.7% of CRMP5-expressing high-grade (WHO grade 3 and 4) tumor cells show PpIX fluorescence. In contrast, CRMP5-expressing low-grade (WHO grade 2) tumor cells do not fluorescence after 5-ALA administration. Furthermore, PpIX fluorescence was not present in areas with intact BBB as assessed by Glut1 and ZO-1 stainings.^34^

Andrieux et al performed RNA profiling of GBM specimens from the tumor core, rim, and invasive margins and demonstrated that 5-ALA-positive cells at the invasive margin form a transcriptionally distinct population for mesenchymal subtype of GBM cells differing markedly from tumor-core and rim regions. 5-Aminolevulinic acid-positive cells contained a mixture of malignant and myeloid-lineage cells. Integration with independent spatial-transcriptomics datasets confirmed that this 5-ALA signature localizes specifically to infiltrative, MRI-invisible regions, and analysis of large clinical cohorts showed that enrichment of the 5-ALA positive gene signature correlates with poor survival and tumor recurrences.^35^

Another study by Lang et al assessed whether 5-ALA fluorescence correlates with local immune-cell infiltration using transcriptomic data. 5-Aminolevulinic acid-related gene expression signature, which was previously described by Bekelis et al was positively correlated with immune-marker RNA levels, namely CD8, CD68, CD168, and FoxP3. Further analysis on 508 immunostained tissue specimen that were acquired intraoperatively did not show consistent ­differences in immune-cell densities using the above-mentioned immune-markers, across regions classified intraoperatively as strongly fluorescent, weakly fluorescent, or nonfluorescent.^36^

Another study that has extensively assessed PpIX localization in GBM specimens is by Nasir-Moin et al who have combined the technology of stimulated Raman histology (SRH) with PpIX spectroscopy to correlate histologic identity with fluorescent and biochemical signatures. One hundred and fifteen patients with (suspected) high-grade glioma were included and fluorescing, and nonfluorescing specimen were acquired during FGS using 5-ALA and then later assessed. This group demonstrated that fluorescence intensity does not correlate with cellularity. Distinct patterns of PpIX accumulation in the intra- and extracellular space were identified. Overall, there was a poor overlap of PpIX fluorescence with the distribution of malignant cells but more with the pattern of myeloid cells, specifically CD163+ macrophages. Furthermore, fiber-like and diffuse bright PpIX fluorescence patterns were correlated with GFAP+ astrocyte-like tumor cells. Also, they demonstrated high co-localization of 5-ALA metabolism, as assessed by heme oxygenase 1 (HMOX1) with immune active regions, that is, mesenchymal-like malignant cells with CD163+ immunosuppressive macrophages. Within the tumor infiltration zone, malignant cells made up 5% of the PpIX-positive cells with the majority being tumor-associated macrophages and oligodendroglial cells.^39^

Single-cell RNA optical RNA optical phenotyping and expression sequencing (SCOPE-seq2) performed by Liu et al on human GBM tissue specimen demonstrated PpIX fluorescence in neoplastic as well as nonneoplastic cells within the TME, but not outside tumor tissue. PpIX fluorescence intensity were comparable between neoplastic cells and oligodendrocytes, but most pronounced in myeloid cells. T-cells showed the weakest PpIX fluorescence among all cells. Further in vitro experiments were performed to assess the mechanism of PpIX fluorescence in nonneoplastic cells. For this purpose, the neurosphere cell line TS543 and the microglial cell line HMC3 were incubated with 5-ALA, both showing PpIX fluorescence. To further characterize PpIX fluorescence mechanisms, both cell lines were incubated with the supernatant of 5-ALA treated TS543 cells. This revealed PpIX fluorescence in both, untreated TS543 and HMC3 cells. Further experiments on ex vivo GL261 mouse glioma slice cultures exhibited stronger PpIX labeling in tumor tissue than in adjacent normal brain, although PpIX signal was detected in both, neoplastic and nonneoplastic cells.^37^

Lastly, a case report on a recurrent atypical meningioma reported 5-ALA-induced fluorescence of the tumor as well as adjacent nonneoplastic gliotic brain tissue. Subsequent histopathological analysis of the fluorescing adjacent gliotic brain tissue revealed reactive gliosis with (chronic) inflammation with CD3- and CD68-positive cells with the latter being the more predominant type.^38^

#### Subcellular localization

Two studies focusing on subcellular localization of PpIX in glioma were identified. The first study demonstrating subcellular localization of PpIX was by Jones et al. U87-MG and U373-MG cells lines were treated with 5-ALA with subsequent isolation of extracellular vesicles (EVs) which showed 247-fold higher PpIX fluorescence compared to controls. These findings were further corroborated in an in vivo xenograft mouse model. This group then determined PpIX-positive EVs in the plasma of patients in GBM patients. Strong intraoperative PpIX fluorescence correlated with increased circulating PpIX-positive EVs compared to pre-5-ALA dosing.^45^

Fredericks et al performed in vitro experiments to further characterize PpIX localization in U118MG cells. They demonstrated that PpIX mainly localizes in the autophagy-lysosomal compartment and only has a weak correlation with the mitochondria. Furthermore, autophagy induction using Spermidine showed increased PpIX fluorescence intensity compared to controls as assessed via flow cytometry.^46^

#### PpIX localization in non-CNS tumor entities

Studies on other tumor entities have also been described regarding 5-ALA uptake and PpIX accumulation and primarily focus on subcellular localization. First experiments by Gaullier et al demonstrated that endogenously produced PpIX mainly accumulates at the plasma membrane and lysosomal structures.^40^ Chen et al demonstrated PpIX accumulation in JCS leukemia cell line using fluorescence spectroscopy. To further assess this, confocal laser scanning microscopy was performed to localize PpIX with organelle-specific fluorescent probes. Confocal images showed diffuse PpIX in cytoplasm and more interestingly, mitochondrial location by showing an overlap of PpIX fluorescence with mitochondrial markers in confocal images.^41^ Ji et al compared PpIX localization patterns in esophageal cancer cell lines after incubation with 5-ALA or purified PpIX using fluorescence microscopy in combination with subcellular markers. They demonstrated that endogenously produced PpIX (via precursor 5-ALA) mainly accumulates in the mitochondria while exogenously applied PpIX demonstrates a diffuse cytoplasmatic distribution and on cell membranes.^42^ Similar results were acquired by Chen et al on follicular lymphoma DHL cells, showing that 5-ALA-induced PpIX accumulation was mainly localized in the mitochondria and endoplasmic reticulum and at lower concentration in lysosomes using 2-photon excitation fluorescence microscopy.^43^ These findings were further corroborated by Wang et al who also demonstrated that endogenous PpIX mainly localizes in the mitochondria while exogenous PpIX localizes predominantly in the cytoplasm and the plasma membrane in lymphoma cell lines.^44^

## Discussion

This review provides a comprehensive overview over current evidence on the cellular and subcellular localization of PpIX accumulation in brain tumors and gives an insight into other tumor entities. Extensive analyses of human GBM specimens using stimulated Raman histology combined with PpIX spectroscopy have shown that fluorescence intensity does not correlate with cellular density but rather that PpIX accumulates in distinct intra- and extracellular patterns.^39^ Stimulated Raman histology produces digital, H&E-like images of unprocessed tissue using intrinsic Raman scattering signals, providing near-real-time histological detail.^47^ Hollon et al demonstrated that deep learning-assisted SRH achieves diagnostic accuracy comparable to frozen sections within minutes.^48^ Subsequent studies confirmed its reproducibility and regulatory approval. When integrated with 5-ALA fluorescence and PpIX spectroscopy, SRH can correlate histologic identity with fluorescent and biochemical signatures. This allows mapping of PpIX-positive tumor cells and TME components (microglia, macrophages, astrocytes) across spatial gradients of fluorescence and infiltration.^39^^,^^47^^,^^48^ Across large surgical datasets, PpIX fluorescence closely aligns with the distribution of myeloid populations, specifically CD163-positive, macrophages, and appears in fiber-like and diffuse patterns associated with GFAP-positive astrocyte-like tumor cells. High colocalization of PpIX metabolism (assessed through HMOX1 expression) is observed in immune-active regions with mesenchymal-origin malignant cells and immunosuppressive macrophages. Within the infiltrative margin, PpIX-positive cells are mainly TAMs or oligodendroglial cells.^39^ The mechanisms underlying distinct PpIX accumulation in specific tumor-associated cell populations remain incompletely understood but may involve differences in cellular heme biosynthesis activity or transporter expression. The influence of any strategies targeting these populations, based on their propensity for PpIX accumulation, specifically on tumor proliferation or immune response, remains unclear.

Single-cell optical phenotyping and transcriptomic profiling demonstrated that PpIX fluorescence appears in neoplastic and non-neoplastic TME cells, with the strongest fluorescence arising from myeloid cells and the weakest from T cells.^37^ In vitro analyses demonstrate that both glioma neurosphere cultures and microglial cells accumulate PpIX following incubation with 5-ALA, as well as the supernatant obtained from initially treated culture.^37^ These findings suggest PpIX fluorescence within the TME may not only result from direct cellular uptake of 5-ALA and conversion to PpIX but also indirect uptake of PpIX released by neoplastic cells. In human surgical specimens, only a subset of high-grade tumor cells demonstrated PpIX fluorescence, whereas low-grade glioma cells remain largely nonfluorescent.^34^ In vitro subcellular localization studies indicate that PpIX preferentially accumulates in autophagy-lysosomal compartments rather than mitochondria, and that autophagy induction increases overall fluorescence intensity.^35^^,^^45^^,^^46^ Additional work shows that glioma cells release PpIX-positive extracellular vesicles both in culture and in vivo. Transcriptomic correlation analyses identify a positive association between a previously established 5-ALA-related gene expression signature and immune-marker mRNA levels; however, immunohistochemical evaluation of more than 500 intraoperatively acquired specimens does not reveal consistent differences in immune-cell densities across strongly fluorescent, weakly fluorescent, or nonfluorescent regions.^35^^,^^45^^,^^46^

### Consequences for 5-ALA Mediated Local Tumor Therapies

For FGS, the cellular and subcellular location of PpIX accumulation in tumor tissue is of minor importance since macroscopically perceived fluorescence depends on overall tissue-level concentrations and optical properties. Distribution is sufficiently aligned with tumor location on a macroscopic scale to achieve precise tumor resections.

However, for 5-ALA mediated local tumor therapies, that is, PDT, SDT, or RDT, the location of PpIX accumulation defines the site of oxidative stress and therefore, overall therapeutic efficacy. Studies have demonstrated PpIX accumulation in both, mesenychymal-like GBM cells and CRMP5-expressing tumor cells.^34^^,^^35^ Mesenchymal GBM cells constitute a highly aggressive and therapy-resistant subpopulation, that is preferentially located at infiltrative tumor margins and within perivascular niches.^49^ CRMP5-expressing tumor cells represent a subset of mesenchymal phenotype and similarly localize at infiltrative margins and promote tumor proliferation.^50^ Selective accumulation of PpIX in these cells may enable specific targeting of these otherwise highly resistant cell populations.^49^^,^^50^

Correlative imaging as performed in the study by Nasir-Moin et al could help determine whether porphyrin activation within compartments of the TME indeed augments therapeutic efficacy in ROS-based modalities. Future multimodal protocols should combine spatial imaging, PpIX quantification, and molecular profiling to establish how porphyrin distribution influences local redox biology, immune response, and treatment outcome.

Several studies have demonstrated that PpIX accumulates in immune and nonimmune-cell populations within the TME.^35-37^^, ^^39^^,^^46^ Pronounced PpIX accumulation has been observed in TAMs with immunoregulatory characteristics as well as microglia, and to a lesser extent, T-cells.^37^^,^^39^ Therapeutic targeting of TAMs in glioblastoma has been explored in preclinical models using colony-stimulating factor 1 receptor (CSF1R) inhibitors, based on the abundant secretion of colony-stimulating factor 1 by glioma cells, which promotes TAM recruitment and expansion.^51^^,^^52^ In murine models, CSF1R inhibition has been associated with antitumor effects and prolonged survival; however, these findings did not translate into clinical benefit in a phase II clinical trial.^51-53^

5-Aminolevulinic acid-mediated local therapies, such as SDT, PDT, or RDT, offer the potential to selectively target TAMs within the TME, as PpIX accumulation in this cell population has been consistently reported across studies. The respective energy source, that is, light, ultrasound or ionizing radiation, could therefore, specifically address this cell population and would not target tumor cells only. The effects of 5-ALA mediated PDT on macrophages have been tested on the murine macrophage cell line RAW264.7, demonstrating an increase in pro-inflammatory markers iNOS, CD86 with a concurrent decrease in immunomodulatory markers ARG1 and CD206.^54^ Furthermore, pro-inflammatory factors, such as IL-6, TNF-a, and IL-1ß were increasingly secreted and macrophage phagocytosis was increased.^54^ Specifically in PDT, high levels of PpIX accumulation and therefore, selective destruction of TAM and also, loss of immunomodulatory phenotypes, have been described in vitro and in vivo.^55^^,^^56^

Microglia are also one of the most abundant immune-cell populations within the TME and frequently adopt an immunosuppressive phenotype. Together with infiltrating macrophages, they constitute TAMs in the TME, and studies also demonstrate PpIX accumulation in microglia. This also makes this immune cells population susceptible to 5-ALA mediated local therapy approaches. By contrast, T-cells exhibit the weakest PpIX fluorescence among immune cells.^37^ T-cells are critical for anti-tumor immune response but exhibit compromised activity within the TME due to TGF-ß secreted by malignant cells as well as invasion of regulatory T-cells that inhibit antitumor responses.^30^^,^^31^ Due to significantly lower PpIX accumulation, PDT, SDT, or RDT would not be highly effective in this population of immune cells. Although further studies specifically investigating these effects on the TME are still necessary, there is extensive research and evidence on immunological responses following 5-ALA-mediated PDT. PDT can induce immunological cell death and release of DAMPs; however, induction of these responses critically depends on the subcellular localization of the photosensitizing agent.^57^ This requirement is fulfilled with PpIX. DAMPs promote antigen presentation and subsequent activation of antitumor immune responses. In addition, PDT has been shown to modulate and reprogram TAMs.^54^^,^^55^ Taking into account that TAMs, particularly subset of immunomodulatory phenotype, exhibit PpIX fluorescence, it is most likely that this observation is due to the subcellular localization of PpIX. From a clinical perspective, PDT has a penetration depth of approximately 4-5 mm. This is due to the short life span and short distance of migration of a singlet oxygen and the limited penetration depth of the light, that is, 4-5 mm, at the preferred wavelength range of 630-690 nm, ensuring minimal light absorption by tissue chromophores.^58^^,^^59^ Consequently, PDT requires precise spatial delivery to achieve adequate tumor coverage. Clinically, PDT can be delivered using 2 principal approaches, that is, interstitial or open (intracavitary) PDT. The most commonly described modality is interstitial which involves stereotactic implantation of cylindrical light diffusers into the target volume following meticulous preoperative 3D planning. Due to the limited penetration, multiple optical fibers are typically required to achieve adequate coverage of the whole tumor volume. While the immunological responses described previously have also been reported following interstitial PDT, open PDT may provide an opportunity to more directly address distinct subcellular niches. In open or intracavitary PDT, optical fibers are positioned within the resection cavity following FGS to either address residual tumor volume or the infiltration zone not amenable for resection to preserve functionality. Two approaches for light delivery have been described: either optical fibers are directly positioned within the resection cavity, or an inflatable balloon applicator containing a single optical fiber is used.^60^^,^^61^ The balloon catheter is filled with transparent fluid and then conforms to the shape of the resection cavity, allowing homogenous light delivery to the cavity walls.^61-63^ The therapeutic efficacy of open PDT has been described in smaller case series and prospective clinical trials.^60^^,^^63^^,^^64^ One phase I clinical trial evaluated the balloon catheter system for open PDT following maximal safe resection, after which patients received standard of care chemoraditation.^61-63^ This study demonstrated notable progression-free and longer overall survivals.^61-63^ Nevertheless, immunological responses and changes in the TME specifically following open PDT should be more systematically addressed in future studies.

Sonodynamic therapy on the other hand is still in its early stages, both in the preclinical and clinical setting. Multiple studies have demonstrated tumoricidal effects and prolonged survival of 5-ALA mediated SDT in the preclinical settings.^65-69^ Antiangiogenetic effects have been demonstrated in preclinical studies.^69^ These effects are likely a result of PpIX accumulation also in endothelial cells, rendering them susceptible to sonication and thereby, impairing their ability to form capillary networks. Clinical translation of SDT remains at an early stage, with most ongoing studies limited to phase 1 and 2 trials, focusing on safety and tolerability. An initial study in a small patient cohort suggested increased apoptosis markers following 5-ALA mediated SDT in patients with GBM, accompanied by a reduction of diffusion coefficient values on post-treatment MRI indicating cytotoxic edema.^70^ Another study, employing a variation of SDT with focused ultrasound in combination with an intravenous 5-ALA formulation, observed pharmacodynamic signs of efficacy for SDT.^71^ While these initial findings suggest therapeutic activity, further clinical trials are required. Notably, the specific effects of 5-ALA-mediated SDT on the TME have not yet been investigated in clinical settings.

A study on cultured THP-1 macrophages demonstrated that 5-ALA mediated SDT induced significant macrophage apoptosis and necrosis through enhanced reactive oxygen species generation and loss of mitochondrial membrane potential.^72^ In preclinical models of melanomas, 5-ALA-mediated SDT has been shown to increase intratumoral macrophages exhibiting pro-inflammatory phenotypes while significantly reducing CD163+ macrophages, indicating a shift toward a pro-inflammatory, antitumor phenotype.^73^ Both in vivo and in vitro studies on melanoma show that 5-ALA SDT upregulates macrophage activation markers such as CD80/CD86 and enhances cytokines, including IL-10, IFN-γ, and TNF-α.^72^^,^^73^ These findings suggest that 5-ALA SDT may exert part of its antitumor activity by reprogramming TAMs and promoting dendritic cell activation and antigen presentation. Although these latter findings were demonstrated in THP-1 macrophages and B16F10 melanomas, there is a rational that similar results can be achieved in gliomas. To date, the effects of 5-ALA mediated SDT on TAMs and specific cell death pathways, including immunological cell death, have not yet been systematically investigated in glioma. Additionally, it should be noted that the TME of melanoma and glioma differ substantially. Therefore, the specific effects of 5-ALA mediated SDT within the TME of glioma remain unclear.

## Conclusion

This review provides a comprehensive overview over cellular and subcellular localization of PpIX accumulation following exogenous 5-ALA administration. In glioma, accumulating data indicate that PpIX localizes not only in malignant tumor cells but also in cellular and noncellular components of the TME. Specifically, tumor-associated macrophages and microglia exhibit distinct PpIX accumulation. These observations are further supported by studies on 5-ALA mediated local therapies such as sonodynamic and photodynamic therapy, which demonstrate immunomodulatory and antiangiogenetic effects. These biological effects may be influenced by spatial and cellular distribution of the sensitizing agent. Our findings provide additional biological rational that 5-ALA-mediated local tumor therapies could potentially contribute to the modulation of the tumor microenvironment.

## Lay Summary

This review examines how 5-aminolevulinic acid (5-ALA), ­routinely used to visualize glioma during surgery, is distributed within glioma and their surrounding environment. The aim of this review was to assess current evidence on its localization and implications for local tumor therapies. Evidence indicates that 5-ALA not only accumulates in tumor cells but also in adjacent immune and supportive cells, as well as in distinct cellular ­compartments. These findings highlight the potential 5-ALA-mediated local tumor therapies which may also address the glioma microenvironment.
